# Identifying Rodent Hantavirus Reservoirs, Brazil

**DOI:** 10.3201/eid1012.040295

**Published:** 2004-12

**Authors:** Akemi Suzuki, Ivani Bisordi, Silvana Levis, Jorge Garcia, Luiz E. Pereira, Renato P. Souza, Teresa K.N. Sugahara, Noemi Pini, Delia Enria, Luiza T.M. Souza

**Affiliations:** * Instituto Adolfo Lutz–São Paulo, São Paulo, Brazil;; † Instituto Nacional de Enfermedades Virales Humanas Dr. Julio I. Maiztegui, Pergamino, Argentina

**Keywords:** Araraquara, Juquitiba, PCR, rodent reservoirs, hantavirus pulmonary syndrome, phylogeny, research

## Abstract

We describe the genetic analysis of samples from hantavirus pulmonary syndrome (HPS) patients from southern and southeastern states of Brazil and rodents captured at the presumed site of infection of these patients. A total of 65 samples that were antibody-positive for Sin Nombre or Laguna Negra virus by enzyme-linked immunosorbent assay were processed by nested reverse transcription–polymerase chain reaction (RT-PCR) by using several primer combinations in the M and S genome segments. PCR products were amplified and sequenced from samples from 11 HPS patient and 7 rodent samples. Phylogenetic analysis of nucleotide sequence differences showed the cocirculation of Araraquara and Juquitiba-like viruses, previously characterized from humans. Our genetic data indicate that Araraquara virus is associated with *Bolomys lasiurus* (hairy-tailed Bolo mouse) and the Juquitiba-like virus is associated with *Oligoryzomys nigripes* (black-footed pigmy rice rat).

## Abstract 2

*Bolomys lasiurus* and *Oligoryzomys nigripes* are rodent reservoirs of Araraquara-like and Juquitiba-like hantaviruses, which cause HPS in Brazil.

Hantaviruses are mainly rodentborne viruses of the family *Bunyaviridae* (*1*). Two clinical forms of infections by hantaviruses are known: hemorrhagic fever with renal syndrome (HFRS) in the Old World and hantavirus pulmonary syndrome (HPS) in the American continent (*2**–**4*). Hantaviruses are enveloped, single-stranded, negative-sense RNA viruses, with a genome with three segments, designated small (S), medium (M), and large (L). The S segment encodes the nucleocapsid protein N, the M segment encodes a glycoprotein precursor that is processed into the envelope glycoproteins G1 and G2, and the L segment encodes the RNA polymerase (*5**,**6*).

The hantaviruses that cause HPS are associated with wild rodents species of the subfamily *Sigmodontinae*. They are transmitted mainly by contact or through aerosols of excrete and secretions of infected rodents (*7**–**9*). Person-to-person transmission has been reported in the 1996 outbreak in Argentina, involving the Andes (AND) virus (*10**,**11*). In Chile, this kind of transmission is suggested by clusters of cases in household contacts (*12*).

In Brazil, during the 1980s and 1990s, virologic and serologic studies conducted in humans and urban rodents showed the circulation of a hantavirus related to Seoul virus (*13**–**16*). In 1993, cases of an acute respiratory illness were detected in a family cluster in Juquitiba County, approximately 80 km from São Paulo City, in southeastern Brazil. Three brothers were affected by the infection, and two of them died (*17**,**18*). Necropsy material from one of them allowed the genetic characterization of a new hantavirus, later named Juquitiba (JUQ) virus, by sequencing a fragment of 139 nucleotides (nt) of the M genomic segment G2 encoding region (*19*). During 1995 and 1996, three more cases of HPS were confirmed by enzyme-linked immunosorbent assay (ELISA); one patient was from the central western county of Vilarejo de Castelo dos Sonhos, in Mato Grosso State, and the remaining two patients were from Araraquara and Franca counties in São Paulo State. Molecular studies carried out on samples from those HPS patients identified two novel genetic lineages of hantaviruses, Castelo dos Sonhos (CAS) and Araraquara (ARA) viruses (*20*). In 1998, new cases of HPS were detected: two in Minas Gerais, four cases in Rio Grande do Sul, and five in São Paulo State. Since then, an increasing number of HPS cases have been diagnosed annually in many states of Brazil. By March 2004, 342 HPS cases had been diagnosed on the basis of characteristic clinical syndrome, epidemiologic data, and Ig (immunoglobulin) M and IgG serologic response against Sin Nombre (SN), Laguna Negra (LN), or AND virus antigens by ELISA (*9**,**21*). Some of these cases were also diagnosed by immunohistochemistry. Most of the HPS cases occurred in the southern and southeastern states of Brazil (177 and 113, respectively). Paraná reported the highest number of cases (n = 92), followed by São Paulo (n = 59), Minas Gerais (n = 54), Santa Catarina (n = 50), Rio Grande do Sul (n = 35), Mato Grosso (n = 33), Maranhão (n = 7), Pará (n = 4), Goiás (n = 3), Rio Grande do Norte (n = 1), and Bahia (n = 1) (M. Elkhoury, pers. comm.).

This study describes the genetic analysis carried out on samples from HPS-case patients from southern and southeastern states of Brazil and rodents captured at the presumed site of infection of the human case-patients. The primary aims were to identify the hantavirus lineages causing HPS in that area, because few reports were available on this topic, and to identify the potential rodent host reservoirs because genetic data were not available from hantavirus-positive rodents. Genetic analysis of the nucleotide sequences indicates that ARA and JUQ-like viruses are circulating in the studied area. We report the genetic identification of the putative primary rodent reservoirs for these viruses.

## Material and Methods

### Area of Study

**Figure 1:**
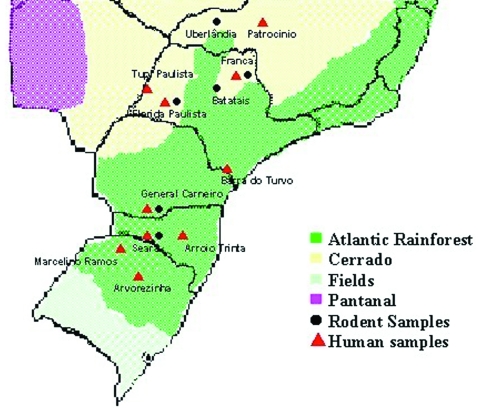
Distribution of natural ecosystems in Brazil. Red triangles and black circles indicate the location of hantavirus pulmonary syndrome cases and rodent capture, which originated the studied DNA sequences, respectively.

The studied areas included two kinds of natural ecosystems: the Atlantic rainforest and "cerrado" at the southern states of Paraná, Santa Catarina, and Rio Grande do Sul and at the southeastern states of Minas Gerais and São Paulo (Figure 1). Basically, the Atlantic rainforest extends along the Brazilian Atlantic Coast, and it is found as umbrofilous tropical forest of hillside or as its regional variation known as Araucaria forest. The cerrado occurs in the Brazilian central plateau and part of northeastern region, and it is characterized by small trees, and grass vegetation, adapted to climates with long dry periods. Both kinds of ecosystems are found in São Paulo and Minas Gerais States.

### Patient and Rodent Samples

We studied samples from HPS patients and from rodents captured at the potential sites where HPS exposures occurred. All samples included in the present study had tested positive to hantavirus by ELISA (*9*) by using SN virus and LN virus antigens (provided by T.G. Ksiazek, Centers for Disease Control and Prevention, Atlanta, GA).

#### Patients

A total of 40 blood and serum samples of HPS patients from five different states of Brazil were processed by nested RT-PCR: 6 samples from Minas Gerais (Patrocinio, Uberaba, Araxá, and Passos); 10 samples from São Paulo (Flórida Paulista, Batatais, Franca, São Carlos, Jaú, Cotia, Barra do Turvo, and Tupi Paulista); 7 samples from Paraná (General Carneiro, Bituruna, Ponta Grossa, Catanduvas, Curitiba, and Guarapuava); 7 samples from Santa Catarina (Seara, Arroio Trinta, and Lindóia do Sul); 10 samples from Rio Grande do Sul (Vacaria, Pelotas, Marcelino Ramos, São Lourenço do Sul, Capão Canoa, Santana do Livramento, Santa Cruz do Sul, Novo Hamburgo, and Arvorezinha). Of seven samples from HPS patients from Santa Catarina, five samples were from a family cluster reported by Seara (*22*).

#### Rodents

Rodents were captured by using Sherman live-capture traps (Sherman Traps Inc., Tallahassee, FL) set in rural or sylvan environments, around the presumed sites of HPS infection. The rodents were processed in the field; biologic samples (blood, liver, kidneys, spleen, heart, and lung) were obtained according to established biosafety guidelines (*23*) and stored in liquid nitrogen for further processing. The carcasses of the rodents were brought to the laboratory; the skins and craniums were used for further identification of the positive specimens. Samples of carcasses were deposited at Museu de Zoologia—Universidade de São Paulo, São Paulo, São Paulo State, and Museum of Southwestern Biology, University of New Mexico, Division of Mammals, Albuquerque, New Mexico, and most of the specimens were deposited in the Vertebrates Collection at Instituto Adolfo Lutz— Seção de Vírus Transmitidos por Artrópodos in São Paulo, SP.

A total of 25 rodent samples (subfamily *Sigmodontinae*) were studied by nested RT-PCR: 3 samples were from Uberlândia (2 *Bolomys lasiurus*) and Uberaba (1 *B. lasiurus*), in Minas Gerais; 13 samples were from Araraquara (1 *B. lasiurus*), Batatais (2 *B. lasiurus)*, Franca (1 *B. lasiurus* and 1 *Calomys tener*), Cassia dos Coqueiros (1 *Oximycterus rutilans*), Cravinhos (1 *B. lasiurus*), Fartura (1 *Akodon* sp.), Mariápolis (2 *B. lasiurus*), Nuporanga (1 *B. lasiurus* and 1 *Oligoryzomys nigripes*), and São Carlos (1 *B. lasiurus*), in São Paulo; 3 samples from General Carneiro (2 *O. nigripes* and 1 *Akodon* sp.) in Paraná; 3 samples from Seara (2 *O. nigripes* and 1 *Bolomys* sp.) in Santa Catarina; 3 samples from Marcelino Ramos (1 *O. nigripes* and 1 *Akodon* sp.) and São Lourenço do Sul (1 *Akodon* sp.), in Rio Grande do Sul.

### RNA Extraction and Nested-RT-PCR

Total RNA was extracted from human blood samples and rodent lung samples by using the RNaid (PLUS) Kit (BIO 101 Inc., La Jolla, CA) as described elsewhere (*24*). Briefly, approximately 100 mg of tissue was mixed with 300 μL of cell lysis solution containing guanidine thiocyanate extracted with phenol/chloroform and purified with RNA matrix beads. From some tissue samples, RNA was extracted with Trizol LS reagent (Invitrogen Co., Carlsbad, CA), following the manufacturer’s recommendations. When human serum specimens were used as source of viral RNA, the QIAmp Viral RNA Kit (Qiagen, Chastworth, CA) was used according to the manufacturer’s instructions. Amplification of virus RNA was performed by "RT-PCR-one step," followed by a second PCR amplification as described previously (*4*). Numerous primer combinations in the M and S segments were used in nested RT-PCR reactions, including oligonucleotide sequences published (*4**,**25**,**26*) and unpublished that were designed to amplify conserved fragments of the S and M genome segments of South American hantaviruses.

### Genetic and Phylogenetic Analysis

The DNA products of the nested PCR reactions were separated from an agarose gel, and bands of the correct predicted size were purified from gel slices with a GeneClean kit (BIO 101 Inc.) or GFXTM PCR DNA and Gel Band Purification Kit (Amersham Pharmacia Biotech, Inc., Piscataway, NJ). The nucleotide sequence of these products was determined on an ABI PRISM 377 Genetic Analyzer (Applied Biosystems, Foster City, CA.) using the dydeoxy cycle sequencing technique (*4*). Sequences were aligned with those of previously described hantaviruses by using BioEdit version 5.0.9 (North Carolina State University, Raleigh, NC) and the computer software package Clustal W 1.4 (*27*). Primer sequences were removed from sample sequences before being aligned. Phylogenetic analysis was carried out on the multiple nucleotide and amino acid sequence alignments by using maximum parsimony (PAUP* version 4.0b4a Macintosh computer software programs) (*28*) and the distance-based neighbor-joining method. Phylogenetic analysis by maximum parsimony was obtained by the heuristic search method. Pairwise genetic distances were computed by using the Kimura-2 parameter, as implemented in the computer program MEGA version 2.1 software (*29*). The bootstrap support for the results of the phylogenetic analysis was based on 500 replicates. GenBank accession numbers of the previously published sequences of the hantaviruses used in this study are listed in figure legends.

## Results

**Figure 2:**
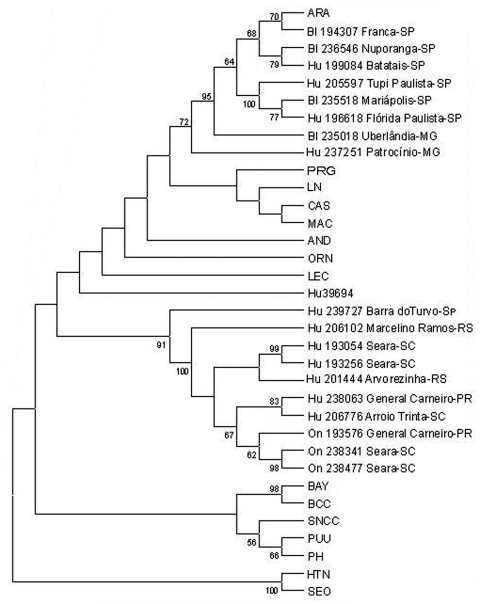
Phylogenetic relationships among Brazilian and previously characterized hantaviruses. Maximum parsimony analysis of the nucleotide sequence of 303-nt fragment of the G2 gene was performed with the heuristic search option. Bootstrap values of >50%, obtained from 500 replicates of the analysis are shown. Abbreviations and GenBank accession numbers of the previously published sequences of the hantaviruses used in this study: Andes, AND-AF324901; Araraquara, ARA-AF307327; Bayou, BAY-L36930; Bermejo, BMJ-AF028025; Castelo dos Sonhos, CAS- AF307326; Hu39694-AF028023; Lechiguanas, LEC-AF028022; Laguna Negra, LN-AF005728; Maciel, MAC-AF028027; Oran, ORN-AF028024; Pergamino, PRG-AF028028; Prospect Hill, PH-X55129; Puumala, PUU-X61034; Sin Nombre, SN-CC74L33684; Hantaan, HTN strain 76/118-Y00386; Seoul, SEO-M34882; Black Creek Canal, BCC-L399500.

PCR products of the expected size were amplified from 11 of 40 HPS patient samples (Table 1) and 7 of 25 rodent samples studied (Table 2). A 303-nt fragment of the G2 gene was amplified and sequenced (bases corresponding to position 2807 to 3109 of Lechiguanas [LEC] virus). Phylogenetic analysis of nucleotide sequence differences showed the cocirculation of two genetic hantavirus lineages previously characterized from humans only: ARA virus and a genotype compatible with the previously identified JUQ virus. ARA virus sequences were derived from eight samples: from three HPS patients and three rodents (*Bolomys lasiurus*) from the state of São Paulo, and 1 HPS case-patient and one rodent (*B. lasiurus*) from Minas Gerais. Pairwise comparisons of the sequences of ARA virus strains from HPS patients and *B. lasiurus* showed an 85.1%-99.7% nt and 95%-99% amino acid (aa) identity. The viral sequence from the HPS patient Hu237251 from Patrocínio, Minas Gerais, the northernmost location included in this study, was most divergent from the other members of this group (16.7%). The second hantavirus genetic lineage identified was closely related to JUQ virus. Samples from seven HPS patients and three *Oligoryzomys nigripes* from the states of Rio Grande do Sul, Santa Catarina, Paraná, and São Paulo fall into this group (Figure 2).

**Table 1:** Sequenced viral RNA samples and epidemiologic data of hantavirus pulmonary syndrome case-patients, Brazil

Specimen	Age (y)	Onset of symptoms	Sample date	County/State	Outcome
Hu193054	25	Oct 18, 2000	NA	Seara, SC^a^	Survived
Hu193256	22	Nov 5, 2000	NA	Seara, SC	Survived
Hu196618	NA	Mar 5, 2001	NA	Flórida Paulista, SP	Died
Hu199084	39	Apr 8, 2001	Apr 13, 2001	Batatais, SP	Survived
Hu201444	28	NA	May 8, 2001	Arvorezinha,RS	Survived
Hu205597	40	Aug 28, 2001	Sep 2, 2001	Tupi Paulista, SP	Survived
Hu206776	NA	NA	Nov, 2001	Arroio Trinta, SC	Died
Hu237251	22	Jul 1, 2002	Jul 17, 2002	Patrocínio, MG	Survived
Hu238063	39	NA	Aug 12, 2002	General Carneiro, PR	Died
Hu239727	32	Oct 8, 2002	NA	Barra do Turvo, SP	Died
Hu206102	52	NA	Sep 27, 2001	Marcelino Ramos, RS	Died

^a^SC, Santa Catarina; SP, São Paulo; RS, Rio Grande do Sul; MG, Minas Gerais; PR, Paraná; NA, not available.

**Table 2:** Rodent samples hantavirus positive by RT-PCR according to location and original ecosystem, Brazil^a^

Specimen code	Rodent species	Original ecosystem	County/state
On193576	*Oligoryzomys nigripes*	Atlantic rainforest	General Carneiro, PR^a^
Bl194307	*Bolomys lasiurus*	Cerrado	Franca, SP
Bl235018	*B. lasiurus*	Cerrado	Uberlândia, MG
Bl235518	*B. lasiurus*	Cerrado	Mariápolis, SP
Bl236546	*B. lasiurus*	Cerrado	Nuporanga, SP
On238341	*O. nigripes*	Atlantic rainforest	Seara, SC
On238477	*O. nigripes*	Atlantic rainforest	Seara, SC

^a^RT-PCR, reverse transcription–polymerase chain reaction; PR, Paraná; SP, São Paulo; MG, Minas Gerais; SC, Santa Catarina.

**Figure 3:**
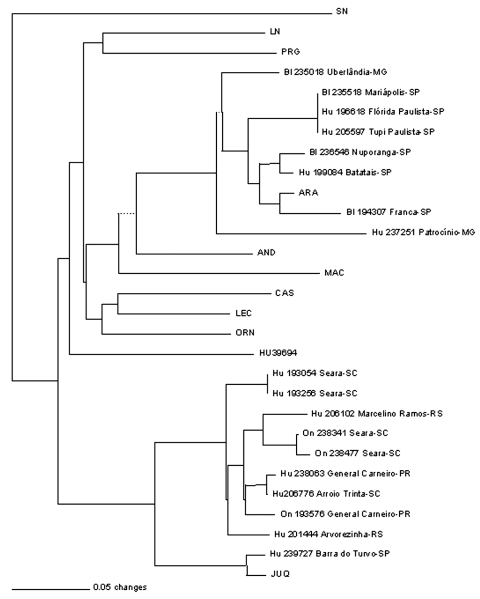
Phylogenetic relationship between newly and previously characterized Brazilian hantaviruses, using a 139-nt region of the M genomic segment G2 encoding region. Maximum parsimony analysis was performed by using the heuristic search option. Bootstrap values of >50%, obtained from 500 replicates of the analysis are shown. Abbreviations and GenBank accession numbers of the previously published sequences of the hantaviruses used in this study: Araraquara-AF307327 and Castelo dos Sonhos-AF307326; Juquitiba, JUQ (*19*).

Sequence comparison with JUQ virus, limited to an overlapping piece of 139 nt of the G2 encoding region, the only available from this virus (19), showed 85.7%-97.7% nt identity (Figure 3). The maximum identity with the prototype JUQ virus was found to correspond to the viral RNA from the patient Hu239727 (97.7%) from Barra do Turvo, São Paulo, the closest location to the site of infection of the fatal HPS case from which JUQ virus was originally characterized in Juquitiba in 1993. Sequences in JUQ-like clade group in two subclades, one including JUQ prototype strain and Hu239727, and the other one comprising six human and the three *O. nigripes* sequences (distance 0.273). The distance observed between these two subclades is intermediate between in-group and between group distances (Table 3).

**Table 3:** Mean distance between sequences^a^

Distance from	ARA clade		JUQ-like clade	
	Mean	SD	Mean	SD
Laguna Negra	0.683	0.037	1.135	0.060
Oran	0.642	0.077	0.785	0.071
Pergamino	0.608	0.019	0.770	0.056
Maciel	0.747	0.042	1.105	0.127
Castelo dos Sonhos	0.555	0.047	0.970	0.046
Hu39694	0.742	0.100	0.723	0.057
Lechiguanas	0.642	0.081	0.798	0.069
Andes	0.516	0.054	0.862	0.044
JUQ-like clade	0.924	0.138	–	–
ARA clade	–	–	0.924	0.138
Inside clade distances	0.149	0.087	0.111	0.089

^a^ARA, Araraquara virus; SD, standard deviation; JUQ, Juquitiba virus.

Comparison of the 303-nt G2-encoding region sequences derived from HPS-patient and *O. nigripes* samples of this group showed 86.6%-99.7 % nt identity. Sequence comparison with the corresponding to ARA group showed 20.8% nt and 4.9% amino acid (aa) divergence. Genetic distances between the sequences studied are shown in Table 3. The general time reversible model (GTR) with a 0.2479 proportion of invariable sites and γ = 0.4121, was used in analysis. AND virus resulted in ARA closest sequence (mean distance = 0.516) and Hu39694, JUQ-like closest sequence (0.723). Mean distances between ARA and JUQ-like sequences further support that they are different viruses.

**Figure 4:**
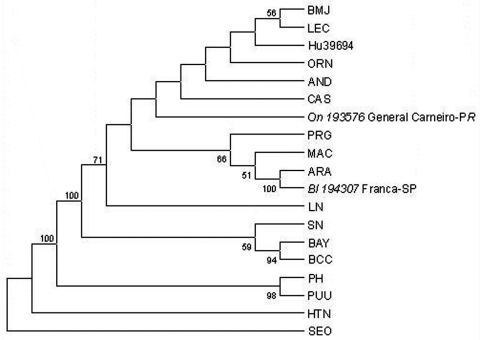
Phylogenetic relationships between Brazilian and previously characterized hantaviruses. Abbreviations and GenBank accession numbers of the previously published sequences of the hantaviruses used in this study are listed in the legend of Figure 2. Maximum parsimony analysis of the nucleotide sequence of the 1,239-nt fragment of the M segment with the heuristic search option. Bootstrap values of >50%, obtained from 500 replicates of the analysis are shown.

A longer fragment of 1,239 nt of the G2 encoding region of the M segment (Figure 4), as well as a fragment of 259 nt of the nucleoprotein encoding region of the S segment, was generated by one representative strain of each hantavirus group (Bl194307 for ARA virus and On193576 for JUQ-like virus). Comparison of viral RNA from *B. lasiurus* sequences with ARA virus showed a 96.8%-nt and 99.3%-aa identity for the 1,239-nt G2-M piece, and 98.8% nt and 100 % aa identity for the S segment piece. When RNA viral sequences from *B. lasiurus* were compared with those from *O. nigripes*, 77.8% nt and 93.4% aa identity for the 1,239-nt G2-M piece, and 84.1% nt and 98.8% aa identity for the S segment piece were observed.

The phylogenetic relationship of ARA and JUQ-like genotypes to other hantaviruses of South America was determined on the nucleotide sequences of the 303-nt and 1,239-nt G2-encoding region of the M segment genomes by using maximum parsimony and neighbor-joining analysis. Both trees showed a similar topology, except for the altered placement of certain genotypes that displayed low bootstrap support. Phylogenetic analysis performed on the 303-nt sequence fragment (Figure 2) showed that all samples from *B. lasiurus* fell into the ARA virus group, whereas all samples from *O. nigripes* fell into the JUQ-like genetic group. The nodes separating these groups had high bootstraps values (72% and 91%, respectively); however, the exact branching order among the Brazilian and the other hantaviruses cannot be resolved by the present phylogenetic analysis. The phylogenetic tree, based on the analysis of the 1,239-nt M segment sequence differences, showed the same topology of the 303-nt sequence, data-based tree. Both maximum parsimony (Figure 2) and neighbor-joining (data not shown) analysis demonstrated that the divergent ARA and JUQ-like viruses form a unique monophyletic clade with the other South American hantaviruses (71% bootstrap support).

Within this clade the ARA virus forms a subclade with other two akodontine-borne Argentinean hantaviruses, Maciel (MAC) from *Necromys benefactus* and Pergamino (PRG) from *Akodon azarae*, although with a low bootstrap support (66%). The JUQ-like virus from *O. nigripes* 193576 is ambiguously placed within the South American viruses, together with CAS virus from Brazil and the rest of the *Oligoryzomys*-borne Argentinean hantaviruses (LEC, Orán, Hu39694, Bermejo, AND virus), as well as LN virus from Paraguay. A slight difference in the topology of this 1,239-nt database tree was observed when neighbor-joining analysis was used; the grouping of this *Oligoryzomys*-borne Argentinean hantavirus clustered in a subclade with a bootstrap support of 79% (data not shown).

## Discussion

Eleven (27.5%) of 40 human samples and 7 (28.0%) of 25 rodent samples studied tested positive for hantavirus by RT-PCR. Previous studies have characterized three different hantavirus genetic lineages associated with HPS cases in Brazil: CAS, ARA, and JUQ (*20**,**30**,**31*).

Previous serologic studies detected IgG antibodies in rodent biologic samples: in *B. lasiurus* and *Akodon* spp. captured in the state of São Paulo (*32*), as well as *B. lasiurus* from São Paulo and Minas Gerais States, and *O. nigripes* from São Paulo, Rio Grande do Sul, Santa Catarina, and Paraná States (*33*).

The data we report on the phylogenetic analysis of viral M and S genome segment fragments, from HPS patients and rodent samples from different locations of southern and southeastern Brazil, showed the circulation of two distinct hantaviruses, closely related to ARA and JUQ viruses, previously characterized only from humans. Our data on the phylogenetic analysis of a 303-nt G2 encoding region of the M genome segment represents the first genetic evidence of the role of *B. lasiurus* as rodent host reservoir for ARA virus, as well as *O. nigripes* as rodent host reservoir for the JUQ-like virus in the region under study. The nodes separating each group of virus within the South American hantaviruses clade were highly supported (72% and 91% bootstrap for ARA and JUQ-like lineages, respectively). The two subclades observed in Figure 3 (i.e., 139-nt tree), and the intermediate distance between them suggested the possibility of some geographic isolation. More extensive sequencing of JUQ prototype and JUQ-like sequences is needed to clarify this point.

Comparison of the sequences of ARA virus strains obtained from rodents (*B. lasiurus*) and HPS patient samples showed an identity of up to 99.7% at the nucleotide level, while within the JUQ-like virus group, the comparison between *O. nigripes* and HPS patient virus strains showed an identity of up to 97.7%. However, the phylogenetic relationship of JUQ-like hantavirus to other members of the South American hantavirus lineages, determined on the nucleotide sequences of the 303-nt sequence, as well as on a longer fragment of a 1,239-nt piece of the M segment performed for one representative strain derived from one *O. nigripes*, systematically failed to resolve the branching order.

In South America, human illnesses associated with hantaviruses have been linked to viruses from the Oryzomyini and Phyllotini tribes. Known akodontine-borne hantaviruses have not been associated with human illnesses. Thus, the data reported here on ARA rodent reservoir constitute the first evidence that a hantavirus associated with an akodontine rodent can cause HPS. Phylogenetic tree based on a 1,239-nt G2 sequence fragment places together the akodontine-borne ARA virus from *B. lasiurus* in Brazil, MAC from *N. benefactus*, and PRG from *A. azarae* in Argentina (*34*). Although the bootstrap support displayed was low (66%), this finding is in accordance with previous observations based on the S genome phylogeny of Argentinean hantaviruses (*35*). This would support the hypothesis of cospeciation of hantaviruses with their specific rodent hosts. As it has been described with other hantaviruses, biogeographic factors are also involved in the evolution of hantavirus lineages (*36**,**37*). The human- and rodent-derived ARA strains analyzed in the current study were distributed at a distance of approximately 650 km. As expected, ARA virus strains originated from the more distant localities displayed the highest genetic divergence, as shown between samples from Patrocínio (Hu237251) and Uberlândia (Bl235018) in Minas Gerais State, and Flórida Paulista (Hu196618) in São Paulo (16.7%-nt difference). Similarly, the divergent JUQ-like virus sample Hu239727 originated from Barra do Turvo, São Paulo, in relation to the rest of the human and rodent JUQ-like virus samples included in this group from locations in Santa Catarina, Paraná, and Rio Grande do Sul may be associated with the geographic distance between them (500 km on average).

Although the data from serologic testing by ELISA indicated four positive *Akodon* spp., one *C. tener*, and one *Ox. rutilans*, specific viral sequences could not be amplified from those specimens. Thus, additional studies are needed to determine the possible role of these species in the epidemiology of hantavirus in Brazil.

The habitats and behavior of the rodents are important aspects to consider in elucidating the reservoirs of etiologic agents. ARA virus was recovered mostly from HPS patients as well as *B. lasiurus* samples from the ecosystem called cerrado, while JUQ-like virus was recovered mostly from human and *O. nigripes* samples from the ecosystem called Atlantic rainforest. Geographic distribution of *B. lasiurus* in Brazil includes the original areas of cerrado and "caatinga." This environment is typical in northeastern Brazil, characterized by deciduous trees and cactus and an extremely prolonged dry season. The *B. lasiurus* distribution shows its ability to adapt to anthropic environments, especially grasses (*Brachiaria*) and sugar cane cultures. These rodents are aggressive and usually dominate the areas they infest (*38*); they do not colonize human dwellings, although occasionally they can invade houses.

*O. nigripes* is adapted to live in the primary and secondary forests, especially in the Atlantic rainforest and Araucaria forest. It is primarily found in anthropic environment, such as the lineal natural habitats bordering cultivated areas, especially those with corn, where it is the most abundant species. These rodents can easily invade dwelling houses and barns to search for food, and they can nest in the domestic habitats.

Among the rodents captured in the cerrado, *B. lasiurus* was the most abundant species (44%) and showed the highest prevalence of antibodies to hantavirus (11%). *Akodon* spp. and *O. nigripes* were the two most abundant among those rodents captured in the transition area between cerrado and the Atlantic rainforest, but the highest prevalence of antibodies to hantavirus was found in *O. nigripes* specimens (8%) (*33*). These data help incriminate *B. lasiurus* and *O. nigripes* in the transmission of ARA and JUQ-like viruses, respectively, to humans.
